# Endogenous growth hormone and insulin after interposition of a reversed jejunal segment in short bowel syndrome. An experimental study on pigs

**DOI:** 10.1186/1756-0500-5-463

**Published:** 2012-08-28

**Authors:** Michail Papamichail, Michail Digalakis, Prigouris Panagiotis, Odysseas Paisios, Soyltana Loti, Theodoros Sergentanis

**Affiliations:** 1 Department of HBP Surgery, Freeman Hospital, Freeman Road, Newcastle, S57AU, UK; 2 General Hospital, Asklipio Voulas, Athens, Greece; 3 General Hospital Evangelismos, Athens, Greece; 4 Medical School, University of Athens, Athens, Greece

**Keywords:** Growth hormone, Insulin, Short bowel syndrome, Reversed jejunal segment

## Abstract

**Background:**

Interposition of a reversed jejunal loop in short bowel sydrome has previously been investigated in human along with animal models and seemed able to facilitate intestinal adaptation. However, it is unclear if growth hormone and insulin, well known for their implication in short bowel pathophysiology, intervene on this effect.

**Findings:**

Porcine models were randomly allocated to two cohorts: (1) short bowel (SB) group (n = 8) and (2) short bowel reverse jejunal segment (SB-RS) group (n = 8). Amongst other parameters serum growth hormone and insulin were measured at baseline, as well as on postoperative day 30 and 60.

**Conclusion:**

Both endogenous hormones failed to demonstrate significant difference in respect to potential direct effect to mechanisms of enhanced intestinal adaptation in reversed group

## Introduction

Regulation of intestinal epithelial loss response is attributed to a complex multistage process with involvement of many different factors [1] including hormones, endogenous intestinal secretions, growth factors and cytokines in immediate interaction with nutrient load and composition [1-3].

A recent meta-analysis has highlighted the promising features of exogenously administered human growth hormone to adult patients with short bowel syndrome [4]. In parallel, the enhancing effect mediated by exogenous oral insulin on intestinal regrowth has been supported by animal models [5].

Our most recent article on the subject [6] consists of an experimental study on pigs; the interposition of a reversed jejunal segment in short bowel animals results suggested to be able to enhance intestinal adaptation at a histopathological level and to favorably modify transit time. Importantly, however, these putatively beneficial actions were not reflected upon body weight [6]. This data builds upon the previous evidence and is a follow-on to this previous research work, assessing endogenous growth hormone and insulin in our experimental setting.

## Material and methods

As described previously6, the pigs were randomly allocated to two groups: (1) short bowel (SB) group (n = 8) and (2) short bowel reverse jejunal segment (SB-RS) group (n = 8). Intestinal transit time and nutritional status (weight and serum albumin) evaluation along with histopathological study and immunohistochemical analysis of small bowel tissue specimens were performed. In addition serum Growth Hormone (Brio 2,Elisa 96,USA) and Insulin (Immulite 1000, DPC,USA) were measured at baseline, as well as on postoperative day 30 and 60. The differences between the two groups were evaluated with Mann–Whitney-Wilcoxon test for independent samples. Statistical analysis was performed with STATA 8.0 statistical software (Stata Corporation, College Station, TX, USA).

## Findings

**Figure 1:**
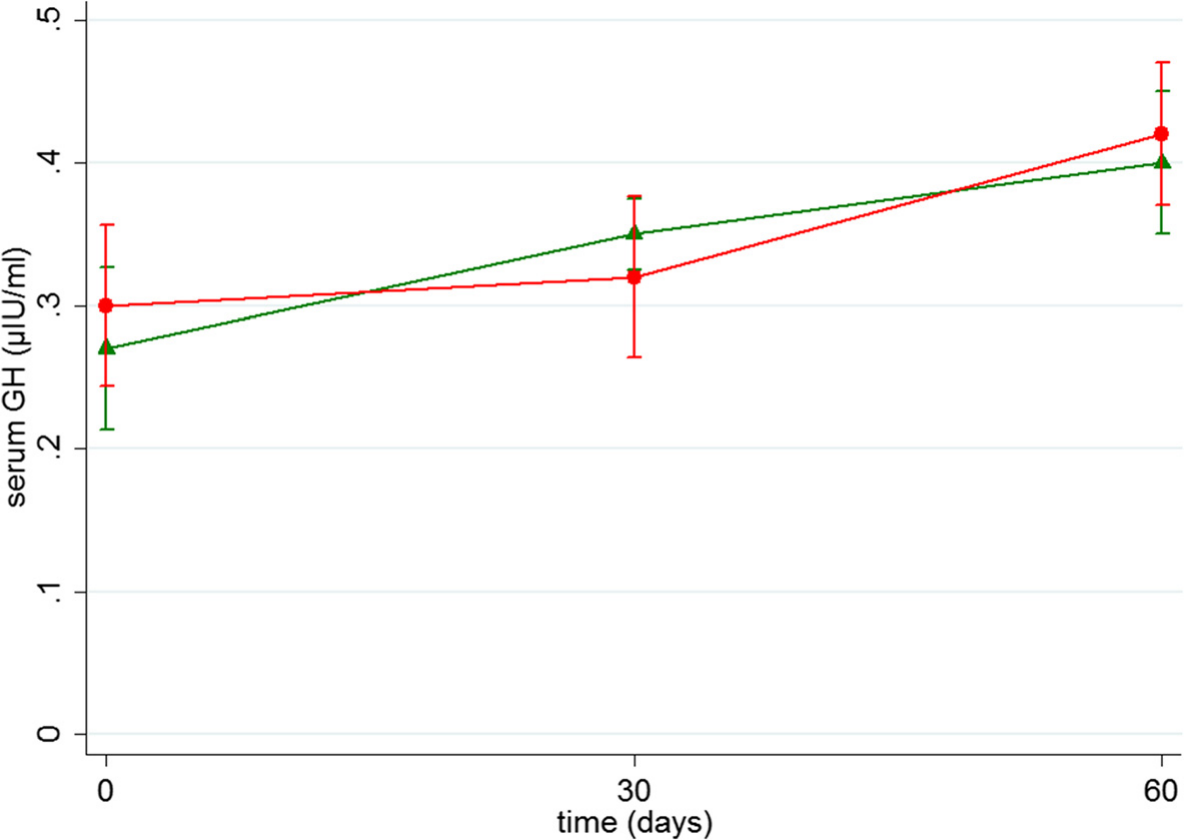
**Diagram demonstrates growth hormone values (μΙU/ml) (mean ± SD) in 2 groups during observation time.** SB group (green) vs. SB-RS group (red).

**Figure 2:**
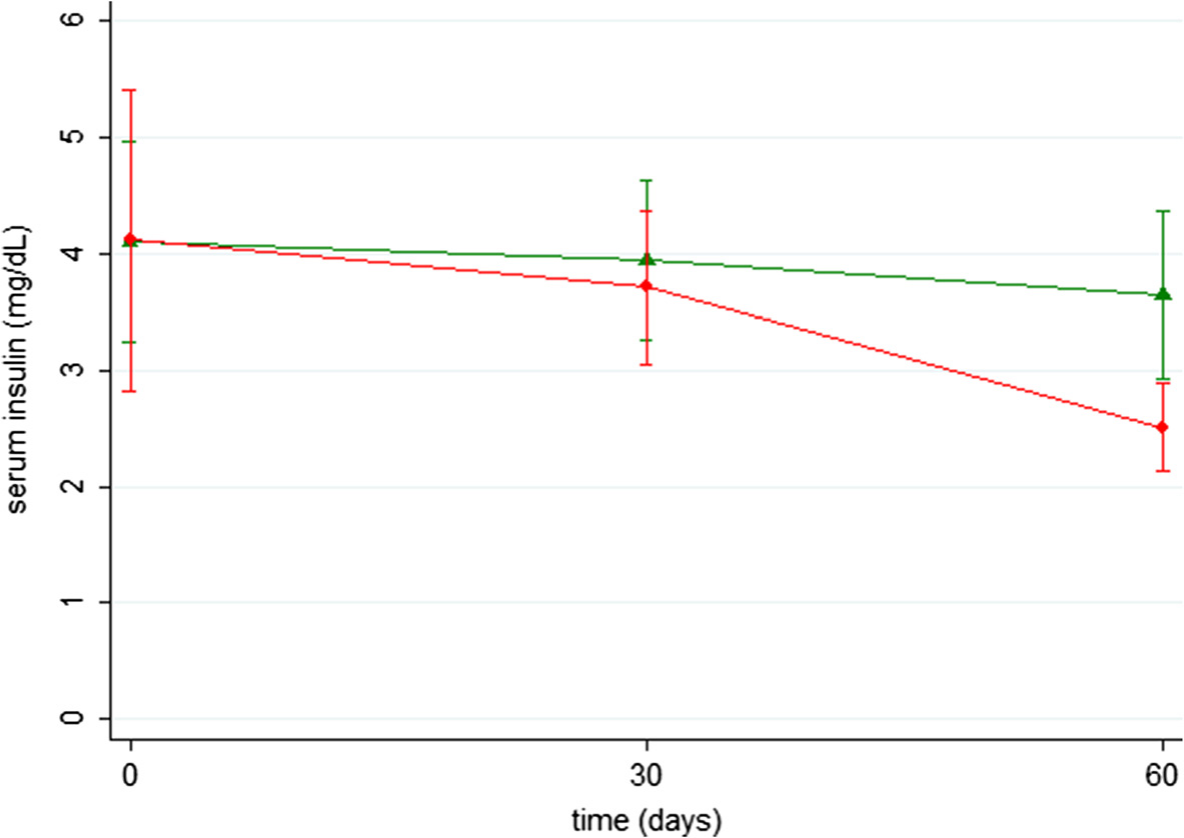
**Diagram demonstrates insulin values (μΙU/ml) (mean ± SD) in 2 groups during observation time.** SB group (green) vs. SB-RS group (red).

No significant differences were demonstrated regarding growth hormone (μΙU/ml) (SB group vs. SB-RS group, mean ± SD: 0.27 ± 0.16 vs. 0.30 ± 0.16, p = 0.562 at baseline; 0.35 ± 0.07 vs. 0.32 ± 0.16, p = 0.672 on postoperative day 30; 0.40 ± 0.14 vs. 0.42 ± 0.14, p = 0.712 on postoperative day 60). Similarly, no significant differences were demonstrated regarding insulin (μΙU/ml) (SB group vs. SB-RS group, mean ± SD: 4.10 ± 2.44 vs. 4.11 ± 3.65, p = 0.495 at baseline; 3.94 ± 1.94 vs. 3.71 ± 1.87, p = 0.833 on postoperative day 30; 3.64 ± 2.03 vs. 2.51 ± 1.07, p = 0.207 on postoperative day 60) (Table 1), (Figures 1,2)

**Table 1:** GH and Insulin values in (μΙU/ml)

	**Group SB**	**Group SB-RS**	**p**
GH baseline	0.27 ± 0.16	0.30 ± 0.16	0.562
GH2 (Post-op D 30)	0.35 ± 0.07	0.32 ± 0.16	0.672
GH final (Post-op D 60)	0.40 ± 0.14	0.42 ± 0.14	0.712
Ins baseline	4.10 ± 2.44	4.11 ± 3.65	0.495
Ins2 (Post-op D 30)	3.94 ± 1.94	3.71 ± 1.87	0.833
Ins final (Post-op D 60)	3.64 ± 2.03	2.51 ± 1.07	0.207

*P values derived from Mann–Whitney-Wilcoxon test for independent samples.

## Discussion

The results suggest a similar outcomes to previous data.The documented difference between two groups, in intestinal growth and proliferation, was attributed at a certain extent on the triggering of the increased decelerated nutrient load [1-3,6]. In several previous studies endogenous insulin and growth hormone have been reported as potent stimulants in intestinal turnover and potentially beneficial factors in the treatment of short bowel syndrome as part of their main anabolic and general regulatory action to overall body metabolic rate and weight balance [7,8]. Actions of GH mediated by Insulin Growth Factor 1 stimulation, resulting in trophic effects in intestinal mucosa in addition with enhancement of intracellular transportation of nutrient constituents [1,7,8]. Similarly insulin is known to be involved in modulation of growth and differentiation of small bowel [5].

The lacks of substantial differences in both factors’ levels appear mainly to reflect the lack of significant weight gain after the interposition in our experimental setting. It may thus be postulated that the histopathologically demonstrated enhanced intestinal adaptation after the interposition [6] and the nutritional status represent two distinct variables which do not obligatorily correlate with each other**.** In parallel with the above it is well known that main source of production of both hormones is other than small bowel and secretion is subject to different stimuli and multifarious regulation systems**.** Indeed, in agreement with the present findings, serum albumin had not exhibited significant changes [6].

Given these findings on endogenous hormones, the future experimental studies should focus on the effect of exogenously administered growth hormone or insulin upon intestinal adaptation after the interposition of a reversed jejunal segment.

## Abbreviations

SB: Short bowel group; SB-RS: Short bowel reverse jejunal segment group.

## Competing interests

Michail Papamichail and other co-authors have no conflict of interest.

## Authors’ contributions

All authors read and approved the final. MP conceived the idea, designed the study and wrote the manuscript. MD conceived the idea, designed the study, revised the manuscript for important intellectual content and has given final approval of the version to be published OP, SL, PP participated in the design of the study, made substantial contributions to the acquisition of data, TNS wrote the manuscript and performed the descriptive statistics.
